# Draft genome sequence of *Stutzerimonas degradans* UO-4-1 isolated from oil-contaminated soil with potential for hydrocarbon degradation

**DOI:** 10.1128/mra.00538-26

**Published:** 2026-06-30

**Authors:** Dong-Eun Lee, Haeun Lee, Dong Jin Choun, Ha Yeon Kang, Dae Hyeon Kim, Gwang Il Jang

**Affiliations:** 1 Department of Dental Hygiene, Ulsan College 65683, Ulsan, Korea; 2 Department of Chemical Engineering, Ulsan College 65683, Ulsan, Korea; University of Southern California, Los Angeles, California, USA

**Keywords:** genome, *Stutzerimonas degradans *UO-4-1, hydrocarbon degradation, oil-contaminated soil

## Abstract

The complete genome of *Stutzerimonas degradans* UO-4-1, isolated from oil-contaminated soil in Ulsan, Korea, spans 4,198,743 bp with a GC content of 64.4% and encodes 3,924 CDs. Functional annotation indicated the presence of enzymes involved in hydrocarbon degradation, including pathways related to the metabolism of aromatic compounds, and fatty acids.

## ANNOUNCEMENT

The genus *Stutzerimonas* was established by reclassifying several *Pseudomonas* species that formed a distinct phylogenetic clade (1, 2). This genus is characterized by broad metabolic versatility and wide ecological distribution, currently encompassing at least 16 validly named species recovered from terrestrial, aquatic, and clinical environments. *Stutzerimonas degradans* UO-4-1 was recovered from oil-contaminated soil in Ulsan (35.31°N, 128.96°E), Korea, using a sterile spatula, and transported to the laboratory in a conical tube. A 1 g soil sample was suspended in 9 mL of phosphate-buffered saline (PBS; Bioneer, Korea), homogenized, and plated onto LB agar (MB Cell, Korea) at 30°C for 48 h. Pure cultures were obtained through three successive streak-plate rounds. For 16S rRNA gene amplification, DNA was extracted from a single colony by the boiling method (3) and used as a PCR template with primers 27F (5′-AGAGTTTGATCMTGGCTCAG-3′) and 1492R (5′-TACGGYTACCTTGTTACGACTT-3′). Purified PCR products were directly sequenced on an Applied Biosystems sequencer (Macrogen, Korea) and compared against the GenBank and EzBioCloud databases using BLASTn (3, 4). After assignment to *Stutzerimonas*, strain UO-4-1 was selected for whole-genome sequencing. A colony was grown in LB broth for 36 h, and cells were harvested from 50 mL of culture via centrifugation. Genomic DNA extraction employed the Maxwell RSC Tissue DNA Kit (Promega, USA) following the manufacturer’s instructions. Library construction used the TruSeq Nano DNA High Throughput Library Prep Kit (Illumina, USA). Briefly, 100 ng of DNA was sheared to approximately 400 bp using adaptive focused acoustics (Covaris), end-repaired, size-selected with magnetic beads, A-tailed, and ligated to indexed adapters before PCR amplification. Library quantity was determined using a quantitative PCR-based kit, and quality was verified with the TapeStation 4200 system with D1000 ScreenTape (Agilent Technologies, USA). Paired-end sequencing (2 × 150 bp) was performed on an Illumina NovaSeq X platform (Illumina, USA) by Macrogen Inc. Genome sequence analyses described hereafter were performed using default parameters for all programs unless otherwise specified. Raw read quality was assessed with FastQC v0.11.7 (5). Reads where more than 10% of bases fell below Q20 were discarded, and adapter sequences were removed with Trimmomatic v0.38 (6). Genome size was estimated from short-read data using Jellyfish v1.1.12 (7) and GenomeScope v1.0 (8). *De novo* assembly was performed with SPAdes v3.15.0 using multiple k-mer values (9), and contigs shorter than 1,000 bp were excluded. Assembly completeness was evaluated with BUSCO v5.1.3 against the bacterial ortholog data set (10). A phylogenetic tree was constructed using MEGA-X 12.0.11 (11).

**Fig 1:**
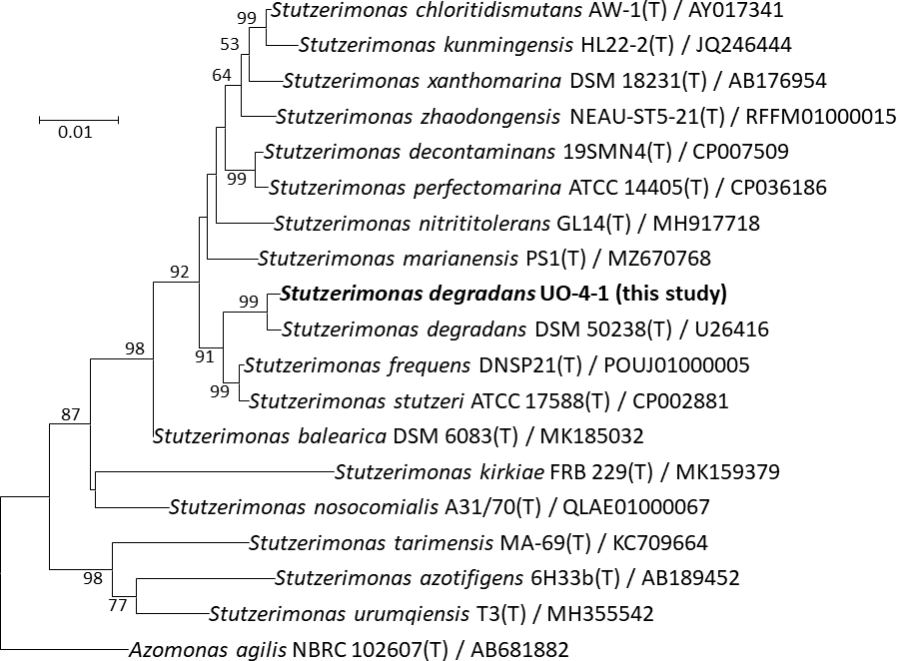
Neighbor-joining tree based on 16S rRNA gene sequences using the Kimura 2-parameter method in MEGA-X 12.0.11, showing the phylogenetic position of strain UO-4-1 within related genera. Bootstrap values were based on 1,000 replicates (values ≥50% were shown). *Azomonas agilis* NBRC 102607(T) was used as the out-group. Bar, 0.01 changes per nucleotide position.

Paired-end sequencing generated 23,770,632 reads, yielding 21,569,664 clean reads (Q20: 99.3%; Q30: 94.9%) at 150.46-fold coverage. The assembled genome spans 4,198,743 bp with a GC content of 64.4%. Annotation identified 3,924 protein-coding sequences (CDSs), 5 rRNA genes, 54 tRNA genes, and 1 tmRNA (Table 1). The 16S rRNA gene is 1,501 bp in length, and phylogenetic analysis confirms placement within the genus *Stutzerimonas* (Fig. 1). The closest relative is *S. degradans* DSM 50238^T^ (GenBank accession no. U26416) at 99.7% sequence similarity.

**TABLE 1:** Information on the draft genome of *Stutzerimonas degradans* UO-4-1

Parameter	Result
Genome size (bp)	4,198,743
GC content (%)	64.40
Total number of contigs	13
Largest contig	1,076,074
CDS	3,924
Number of total genes	3,984
Total number of reads	4,170,936
N50	823,435
tRNAs	54
rRNA	5
tmRNA	1

## Data Availability

The whole-genome sequencing project for *Stutzerimonas degradans* UO-4-1 has been deposited at DDBJ/ENA/GenBank under accession number JBXQXT000000000. The version described in this paper is the first version, JBXQXT010000000. The BioSample accession number SAMN57360976 and SRA accession number SRR38179161 are available on NCBI.
